# One Ostium, Three Coronary Arteries: A Rare Coronary Anomaly in a Patient With Secundum Atrial Septal Defect

**DOI:** 10.7759/cureus.104706

**Published:** 2026-03-05

**Authors:** Anna Joseph, Muhammad A Sultan, Violeta Groudeva, Pencho Kratunkov

**Affiliations:** 1 Cardiovascular Medicine, Medical University Sofia, Sofia, BGR; 2 Internal Medicine, Medical University Sofia, Sofia, BGR; 3 Diagnostic Imaging, University Hospital St. Ekaterina, Medical University Sofia, Sofia, BGR; 4 Congenital Heart Diseases in Children and Adults, University Hospital St. Ekaterina, Medical University Sofia, Sofia, BGR

**Keywords:** atrial septal defect, congenital heart disease, coronary artery anomaly, ct coronary angiography, interarterial lad, single coronary artery

## Abstract

Atrial septal defects (ASDs) are among the most common congenital heart defects diagnosed in adulthood, with percutaneous device closure being the standard treatment for suitable secundum ASDs. However, the presence of anomalous coronary artery anatomy, particularly a high-risk interarterial course, may complicate procedural planning due to potential myocardial ischemia and sudden cardiac death. Single-ostium coronary artery anomalies are exceptionally rare, and their coexistence with a secundum ASD is even more uncommon, providing distinct diagnostic and treatment issues.

We report the case of a 42-year-old woman with a secundum ASD. Her background included obesity, hypertension, and anxiety. Investigations revealed a significant left-to-right shunt (Qp/Qs 1.72:1), raised pulmonary artery pressures, and right-heart dilation. As part of pre-procedural assessment, CT coronary angiography unexpectedly identified a single coronary artery arising from the right coronary sinus. This single ostium gave rise to three branches: a right coronary artery (RCA) with a normal course, a retroaortic left circumflex (LCx), and a left anterior descending (LAD) artery with an interarterial course between the aorta and pulmonary artery. Despite the anatomical anomaly, high-intensity treadmill testing showed no chest pain, electrocardiogram (ECG) changes, or wall-motion abnormalities. Multidisciplinary evaluation confirmed that no coronary intervention was required due to the absence of ischemia. Therefore, percutaneous transcatheter closure was indicated, given the adequate septal rims. The procedure was successfully performed using a 22 mm Amplatzer septal occluder without complications, and complete elimination of the shunt was achieved. Follow-up imaging confirmed normal device position and stability, reduced pulmonary pressures, and preserved coronary artery patency.

This case highlights the critical role of multimodality imaging, in particular CT angiography and functional testing, in the pre-procedural evaluation of coronary anatomy prior to device closure in ASD patients. It emphasizes that in the absence of functional ischemia, even a potentially high-risk single-ostium coronary anomaly may not be a contraindication to safe transcatheter ASD closure. It also underlines the value of a multidisciplinary approach in complex congenital presentations where routine pathways may need to be adapted.

## Introduction

An atrial septal defect (ASD) is a congenital cardiac malformation characterized by a persistent opening in the interatrial septum, allowing abnormal shunting of blood from the left to the right atrium (RA). ASDs are among the most common types of congenital heart diseases (CHDs), accounting for about 10% of all CHDs [1]. The overall prevalence of ASD is approximately 1.6 per 1,000 live births [2].

ASDs are classified into four main types: ostium secundum (ASD II), ostium primum (15%-20%), sinus venosus (5%-10%), and coronary sinus defects (<1%). The most prevalent type is ASD II, which accounts for 70%-80% of cases and is located at the fossa ovalis in the mid-septal region [3].

While some ASDs are diagnosed at an early age, many cases, especially the ostium secundum type, can remain undetected until adulthood, often presenting with non-specific symptoms like fatigue or breathlessness [4]. In particular, an aneurysmal interatrial septum with a large defect can create a significant left-to-right shunt, increasing blood flow to the right side of the heart. Over time, this extra strain can lead to pulmonary hypertension and eventually heart failure if left untreated [5].

By contrast, a single‐coronary‐artery anomaly, where all coronary branches arise from a common ostium, is extremely rare, with an estimated incidence of 0.024% to 0.066% in the general population. These anomalies are typically asymptomatic and often discovered incidentally on imaging [6]. Symptomatic patients may report chest pain, exertional dyspnea, or syncope. Among the various anatomical variants, the interarterial course carries the highest risk due to the possibility of dynamic compression between the aorta and pulmonary artery (PA), which can lead to myocardial ischemia or infarction, malignant arrhythmias, or even sudden cardiac death. Several systems exist to classify coronary artery anomalies. One of the most widely used is the Lipton-Yamanaka classification, which categorizes anomalies based on the sinus of origin (right (R) vs. left (L)) and the distribution pattern (Type I-III), with additional course subtypes: anterior (A), interarterial (B), posterior (P), and septal (S). However, it is important to note that there is no unified classification system for coronary artery anomalies. The most important and widely accepted classifications categorize them based on their morphology and hemodynamic significance. Among these, an alternate and frequently referenced model is Angelini’s classification, which defines coronary anomalies by their morphology, structural pathways, and myocardial distribution [7]. This approach offers a more functional perspective, correlating anatomical variations with their potential clinical consequences and hemodynamic significance.

The presence of an ASD alongside an anomalous coronary artery significantly alters the risk profile and demands advanced imaging and meticulous procedural planning. Fortunately, treatment has evolved beyond open‐heart surgery. Most patients with ASD II now undergo minimally invasive transcatheter closure using devices such as the Amplatzer septal occluder, which have demonstrated excellent outcomes and become the standard approach [8]. However, when a patient has other congenital anomalies, such as a single ostium giving rise to all three major coronaries (right coronary artery (RCA), left anterior descending (LAD), and left circumflex (LCx)), multimodality imaging and heart-team planning are vital to ensure safe device deployment without compromising coronary flow [9].

In this report, we share the case of a 42-year-old woman with ASD II and an aneurysmal atrial septum, who also had obesity, hypertension, and a rare coronary artery anomaly. After thorough imaging and a multidisciplinary approach, she underwent successful transcatheter closure of her ASD.

## Case presentation

A 42-year-old woman presented to her general practitioner with arterial hypertension since 2023, reaching values up to 170/100 mmHg on several occasions, and sertraline (Zoloft) had been initiated because anxiety had been considered the main cause. She had also been consulted with a couple of cardiologists who discovered that she had an ASD and added antihypertensive therapy with Valtricom 5/160/12.5 mg (amlodipine/valsartan/hydrochlorothiazide) daily alongside Zoloft. She also has obesity (BMI = 32.28) and gained weight of 20 kg over the past 18 months, which was considered to be multifactorial. The patient has been referred to our hospital for evaluation of the ASD.

On physical examination, she was alert, cooperative, and hemodynamically stable. Her blood pressure measurements were within the normal range during hospitalization. Cardiac auscultation revealed a grade 2/6 systolic murmur best heard at the second left intercostal space. There were no signs of respiratory compromise, and no peripheral edema was observed. Initial electrocardiogram (ECG) demonstrated sinus rhythm at 64 bpm, without evidence of ischemia or repolarization abnormalities. Baseline laboratory investigations, including renal function and thyroid-stimulating hormone (TSH), were within normal limits.

**Figure 1:**
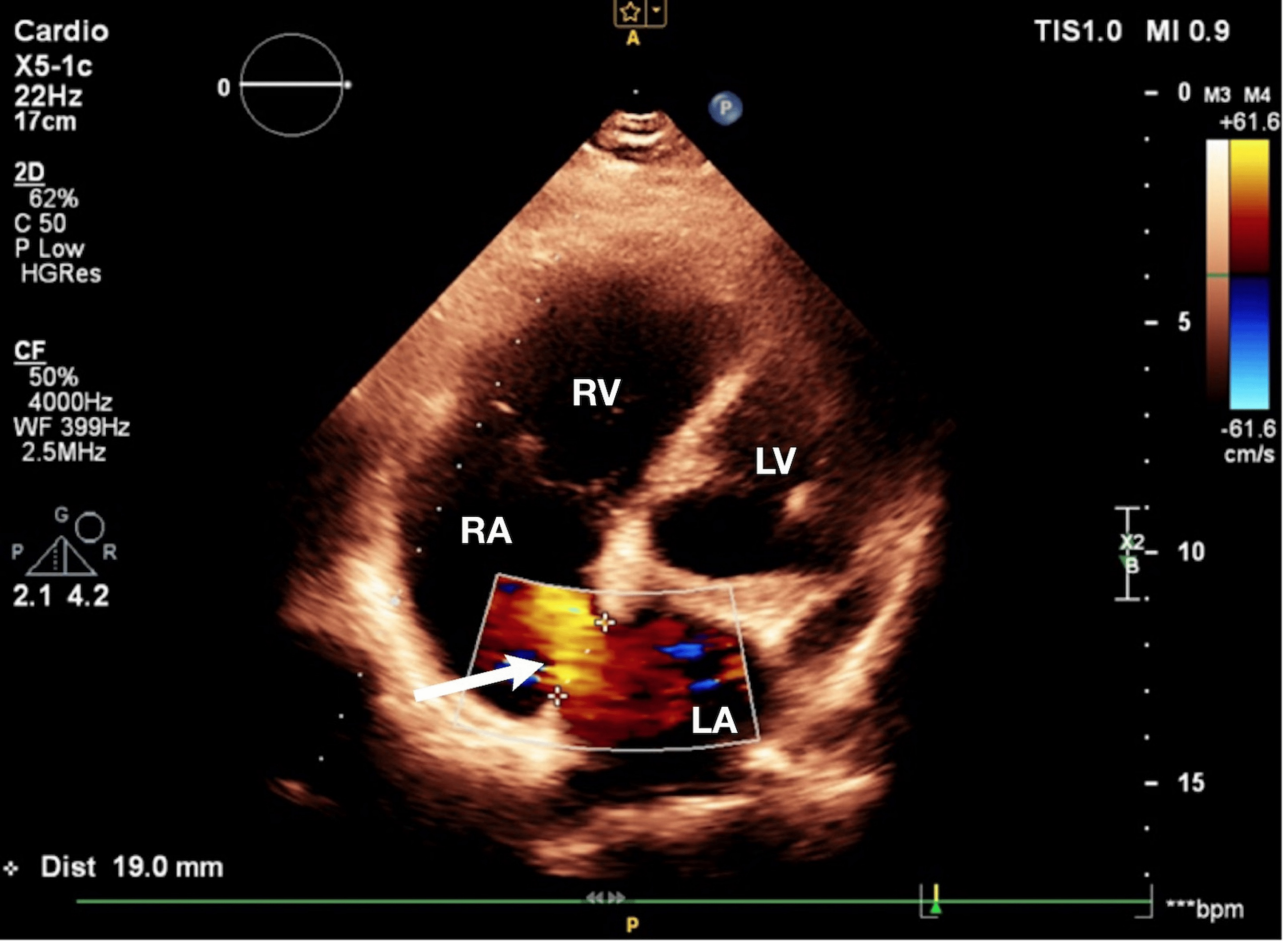
Transthoracic echocardiography with color Doppler demonstrating a secundum atrial septal defect (ASD II) with left-to-right shunting (apical four-chamber view). RV: right ventricle; RA: right atrium; LV: left ventricle; LA: left atrium

**Figure 2:**
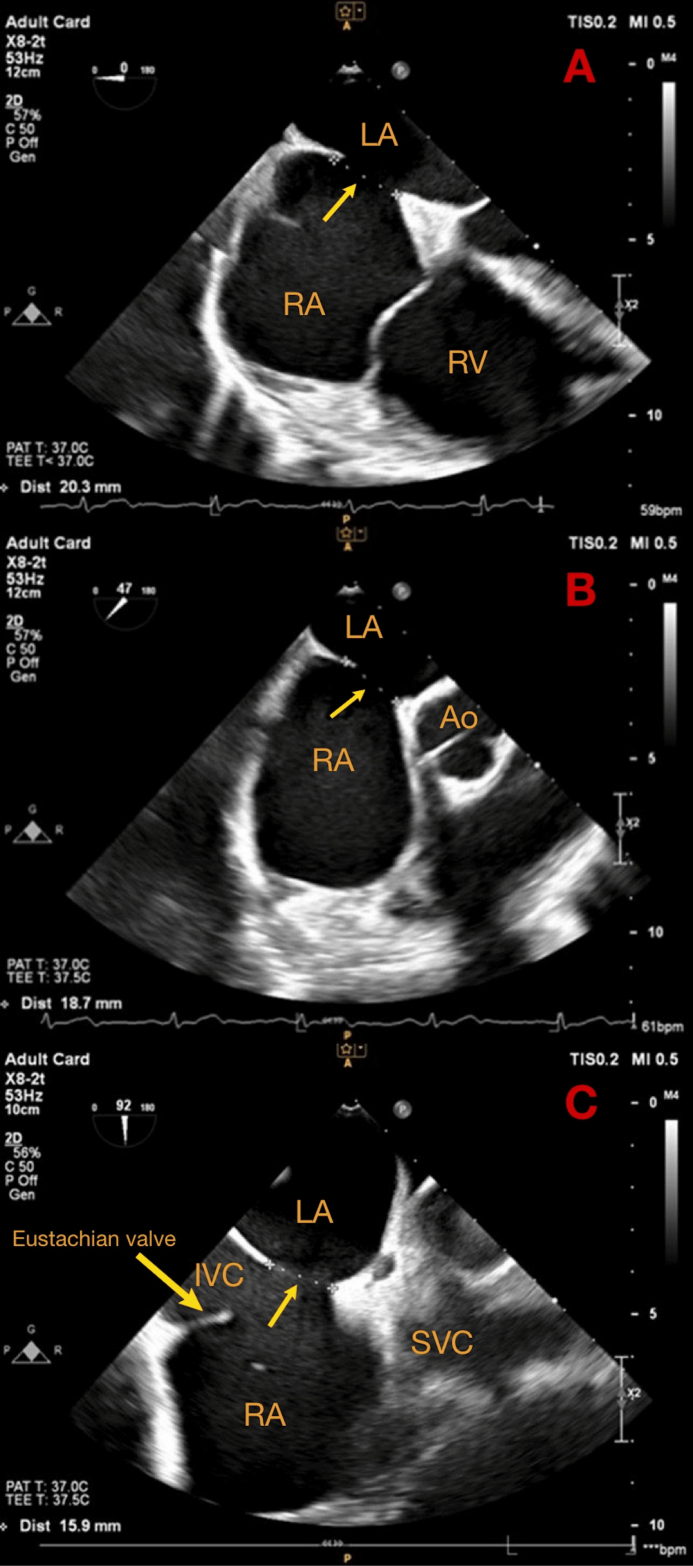
Transesophageal echocardiography (TEE) with four-chamber view (A), short axis view (B), and bicaval view (C) at 0°, 47°, and 90°. RV: right ventricle; RA: right atrium; LA: left atrium; Ao: aorta; SVC: superior vena cava; IVC: inferior vena cava

**Figure 3:**
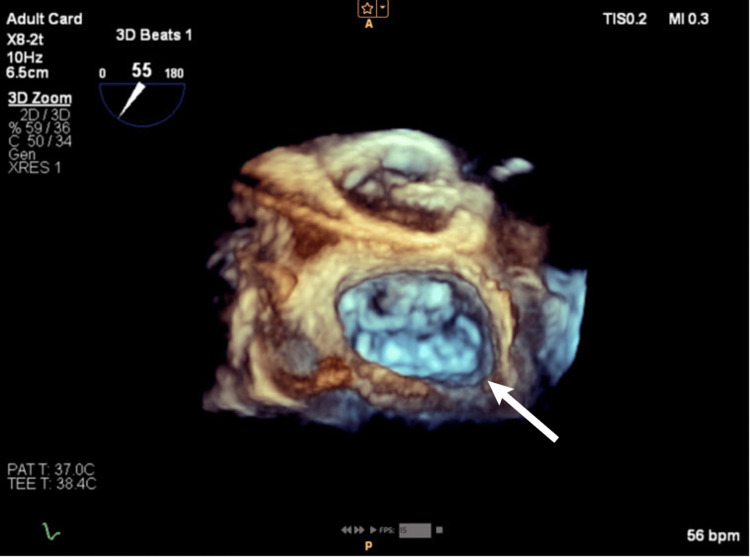
Real-time 3D TEE image of ASD II from the RA perspective. TEE: transesophageal echocardiography; RA: right atrium; ASD II: secundum atrial septal defect

Transthoracic echocardiography (TTE) followed by transesophageal echocardiography (TEE) identified two significant structural findings: a 16 mm ASD II, with left-to-right shunt, and an aneurysmal interatrial septum (Figures 1, 2A-2C, 3). The ASD lacked an anterior aortic rim, but its borders were well formed, making it suitable for device closure. There was mild enlargement of the RA and right ventricle (RV), suggestive of chronic volume overload. PA systolic pressure was estimated to be ~35-40 mmHg, consistent with developing pulmonary hypertension. Both the mitral and tricuspid valves had trivial regurgitation, left ventricular (LV) function was preserved, and there was no evidence of intracardiac thrombus or pericardial effusion.

**Figure 4:**
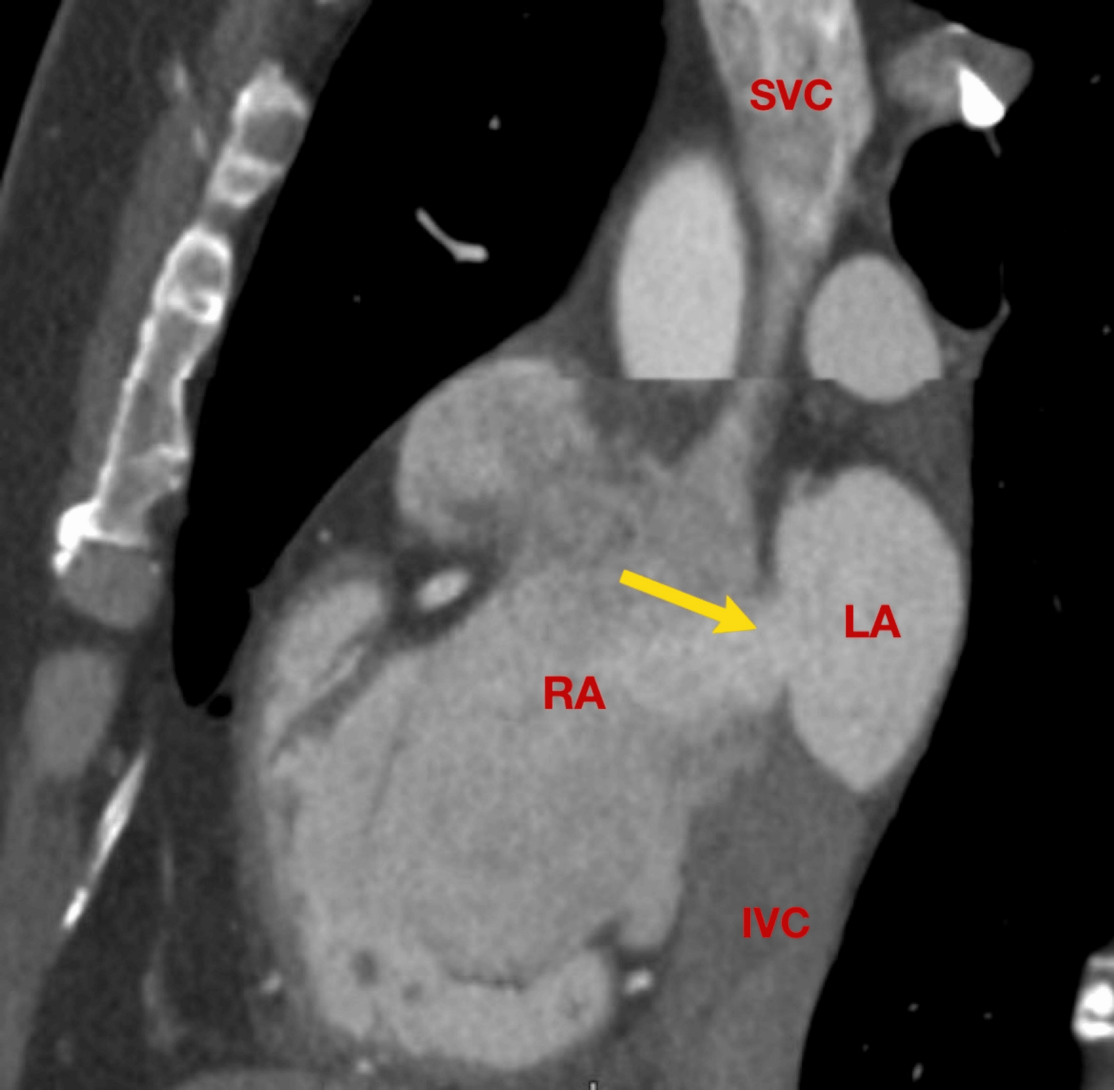
Multidetector CT (MDCT): multiplanar reformat view at the level of the atria, corresponding to the TEE bicaval view, demonstrating the atrial septal defect. TEE: transesophageal echocardiography; SVC: superior vena cava; IVC: inferior vena cava; RA: right atrium; LA: left atrium

**Figure 5:**
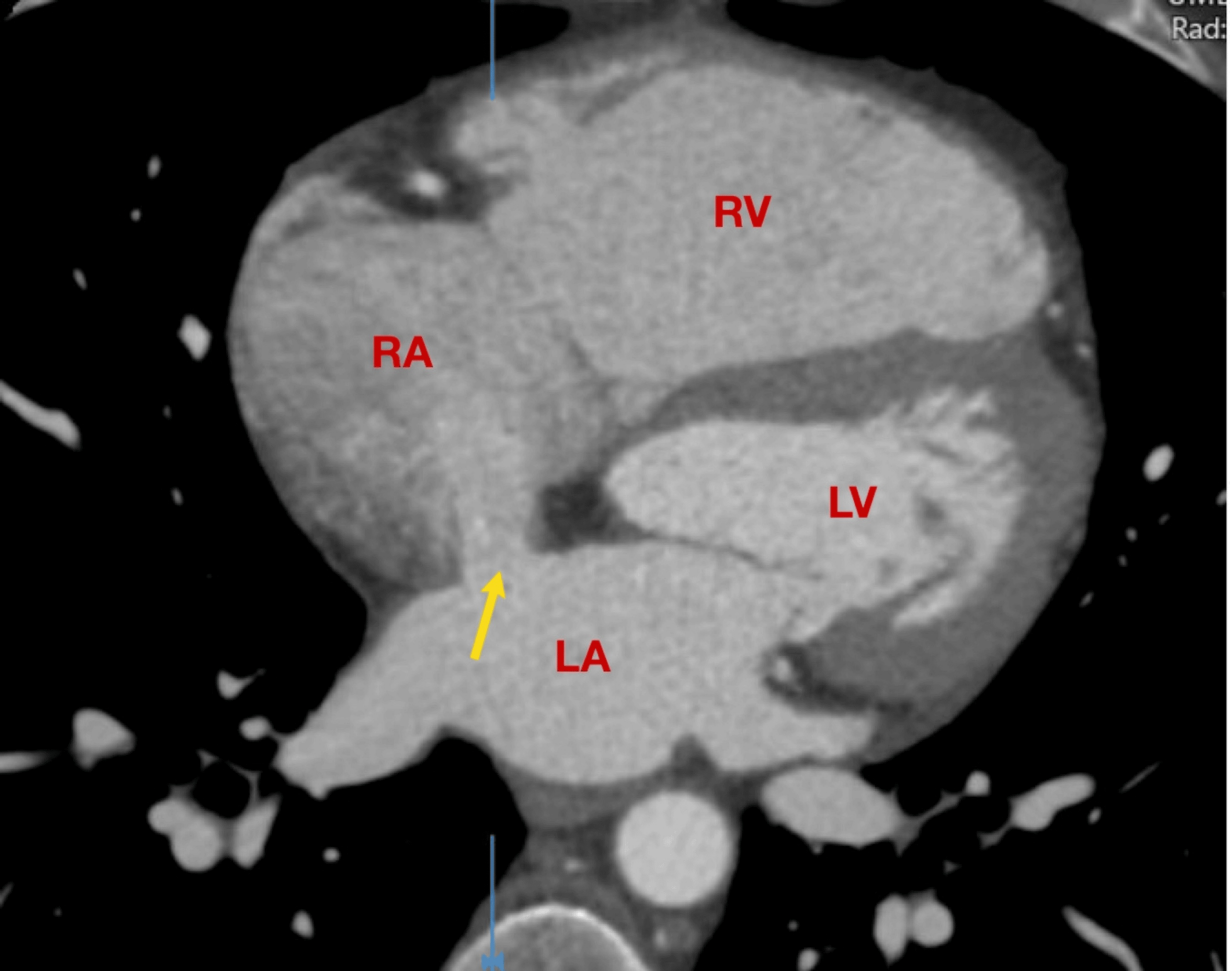
MDCT: four-chamber view demonstrating the atrial septal defect with a jet from the left to the right atrium. MDCT: multidetector CT; RV: right ventricle; RA: right atrium; LV: left ventricle; LA: left atrium

**Figure 6:**
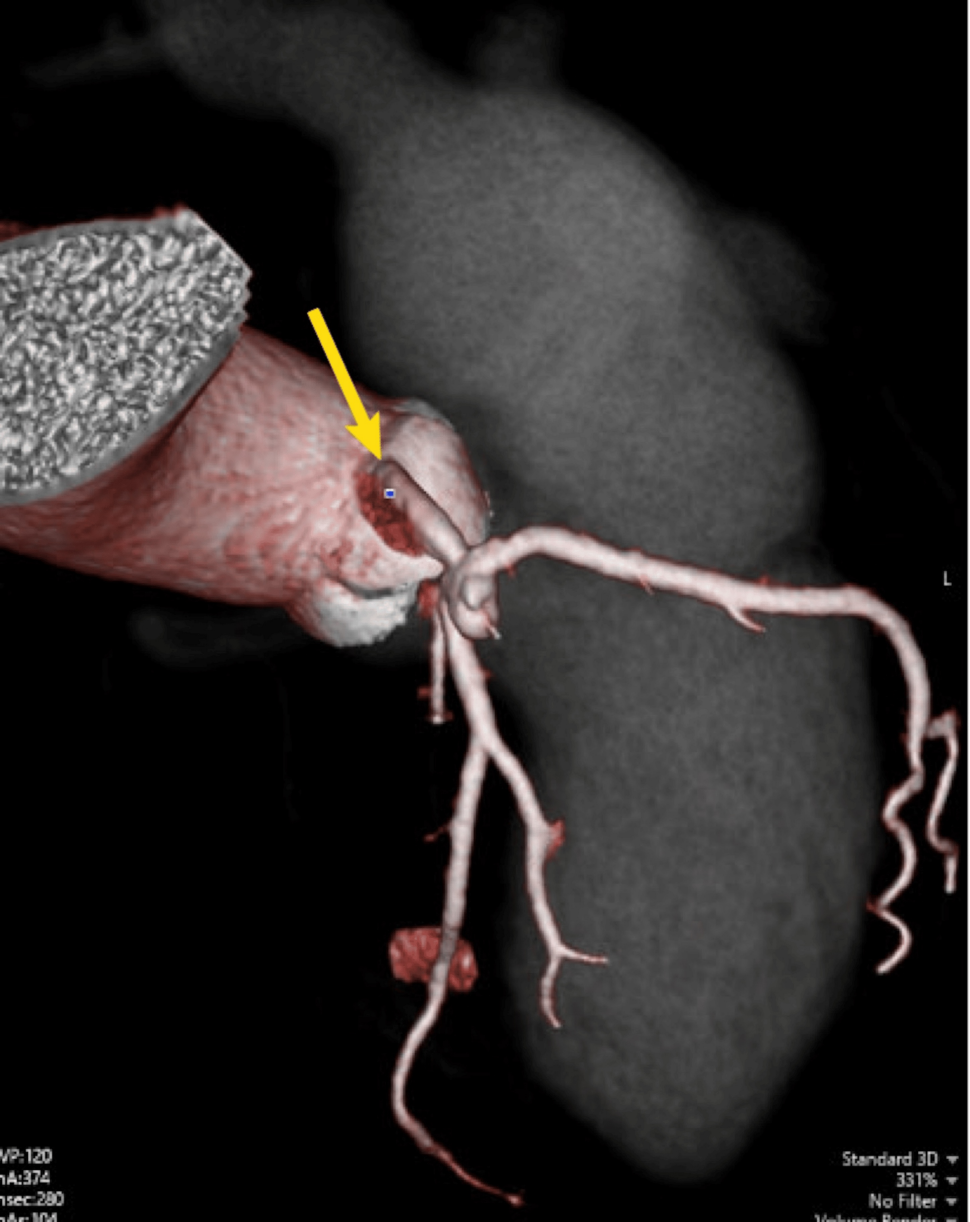
MDCT: volume rendered vessel reconstruction demonstrating all coronary arteries arising from the right coronary sinus. MDCT: multidetector CT

**Figure 7:**
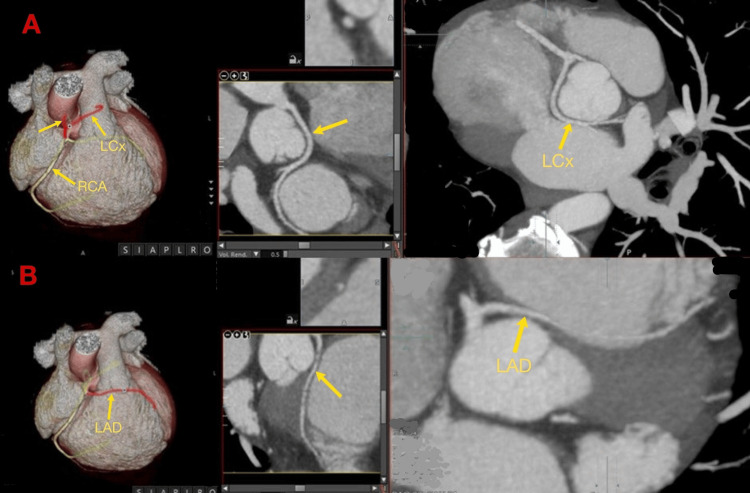
MDCT: demonstrating the origin of the coronary arteries arising from the right coronary sinus-the retroaortic course of LCx (A) and the interarterial course of the LAD (B). MDCT: multidetector CT; LCx: left circumflex; LAD: left anterior descending; RCA: right coronary artery

To support pre-procedural planning and rule out any additional thoracic or vascular abnormalities, a coronary CT angiography (CTA) was performed. Coronary CTA confirmed an ASD II in the fossa ovalis, with well-defined posterior and inferior rims and a missing anterior rim (Figures 4, 5). Multidetector CT (MDCT) showed no evidence of anomalous pulmonary venous return, supporting suitability for transcatheter closure. The interventricular septum was observed to be intact. Right‐heart enlargement was confirmed by a transverse RV diameter of 46 mm versus a LV diameter of 43 mm (RV:LV > 1 reflects chronic volume overload from the left-to-right shunt). Both ventricles were contracting normally and maintained excellent systolic function with LV ejection fraction (LVEF) 82% and RV ejection fraction (RVEF) 58%. The RV end‐diastolic and end-systolic volumes were markedly increased at 180 and 76 mL, respectively, yielding a stroke volume of 103 mL and cardiac output of 7 L/min. These findings are consistent with significant left-to-right shunting. LV myocardium appeared normal, and a small (≤7 mm) basal pericardial effusion was noted, but it was considered hemodynamically insignificant. Contrast CTA also revealed an incidental single coronary artery anomaly with all coronary arteries (RCA, LAD, and LCx) arising from the right coronary sinus through a short common ostium (Figure 6). The RCA follows a normal course in the right atrioventricular groove without stenosis. The LAD takes an interarterial route between the right ventricular outflow tract (RVOT) and the aortic root before entering the anterior interventricular groove, with no luminal narrowing. The LCx follows a retroaortic course in the atrioventricular groove, also without stenosis (Figure 7).

A cardiac stress test was performed using a modified Bruce protocol to assess for inducible ischemia due to the patient’s coronary anomaly. She exercised for a total duration of eight minutes, reaching stage III with a peak heart rate of 164 bpm and blood pressure of 120/60 mmHg. No chest pain, ST-T changes, or significant arrhythmias were observed, apart from isolated ventricular extrasystoles in early recovery. These findings demonstrated good physical capacity and preserved coronary reserve.

**Figure 8:**
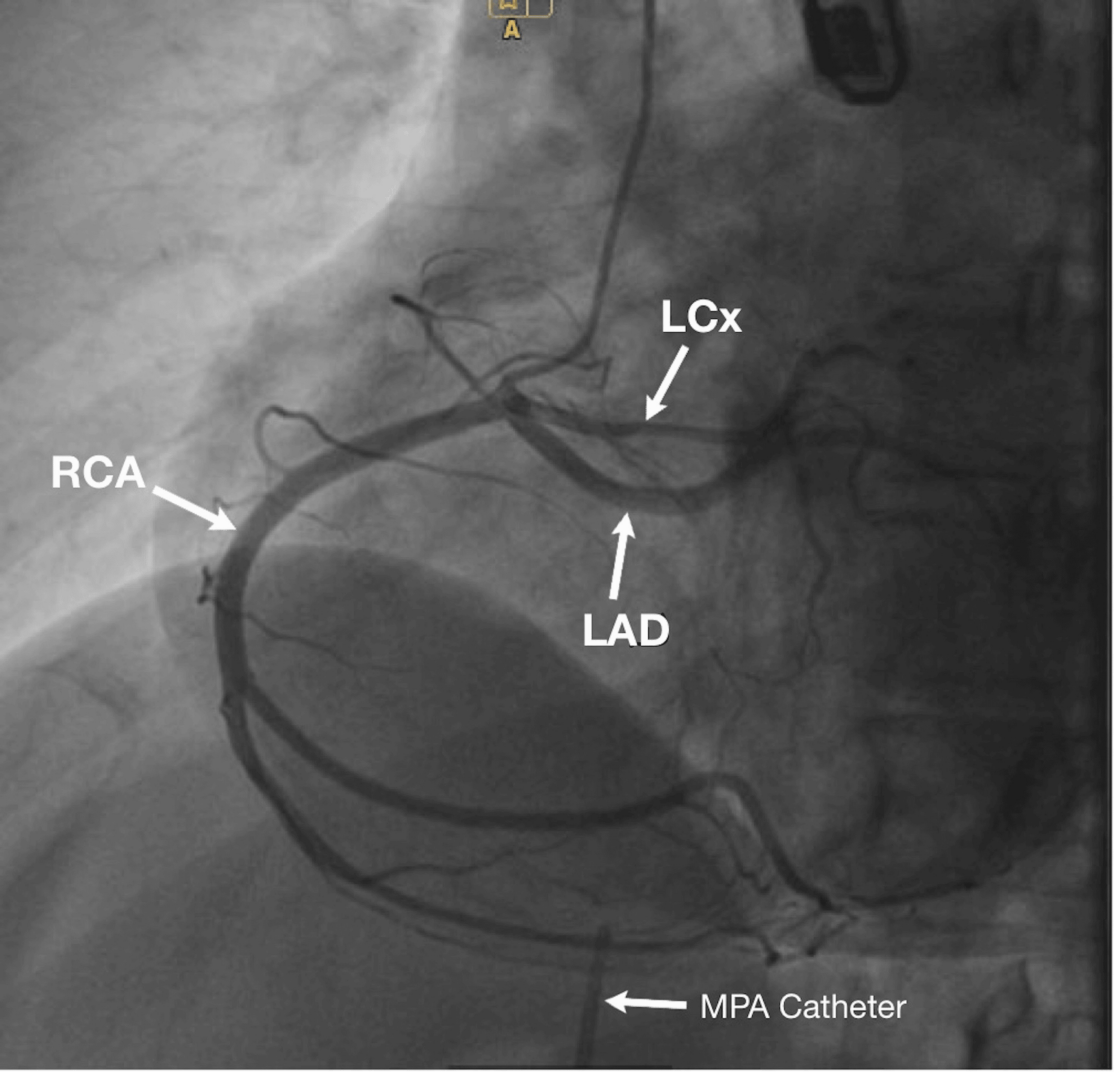
Coronary angiography demonstrating a single coronary ostium arising from the right coronary sinus (anteroposterior view). LCx: left circumflex; RCA: right coronary artery; LAD: left anterior descending; MPA: multipurpose angiographic

**Figure 9:**
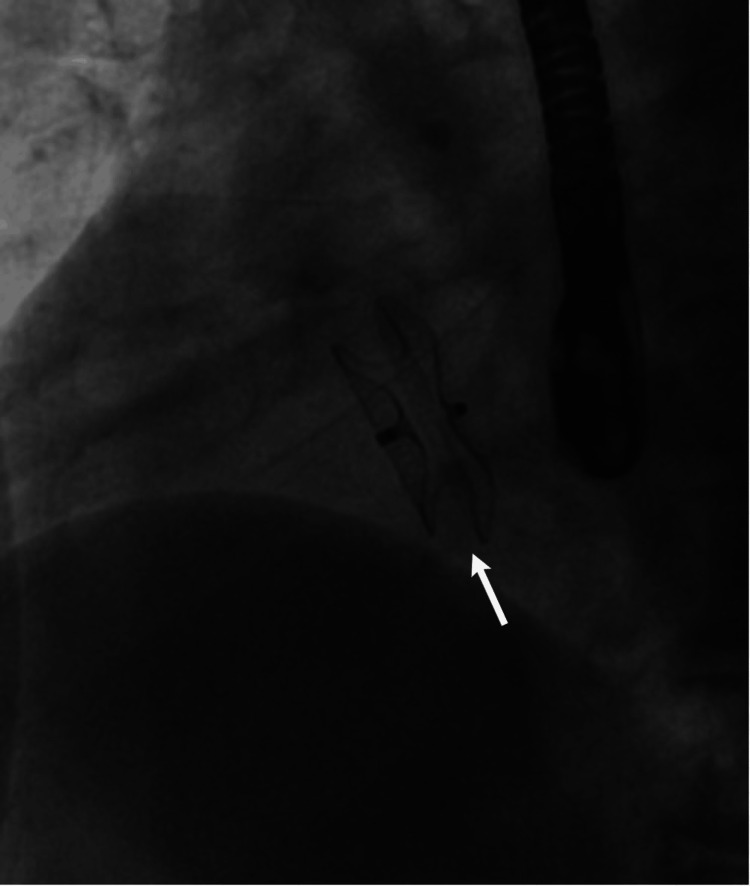
Fluoroscopic image demonstrating the Amplatzer septal occluder (ASO) securely deployed across the interatrial septum.

**Figure 10:**
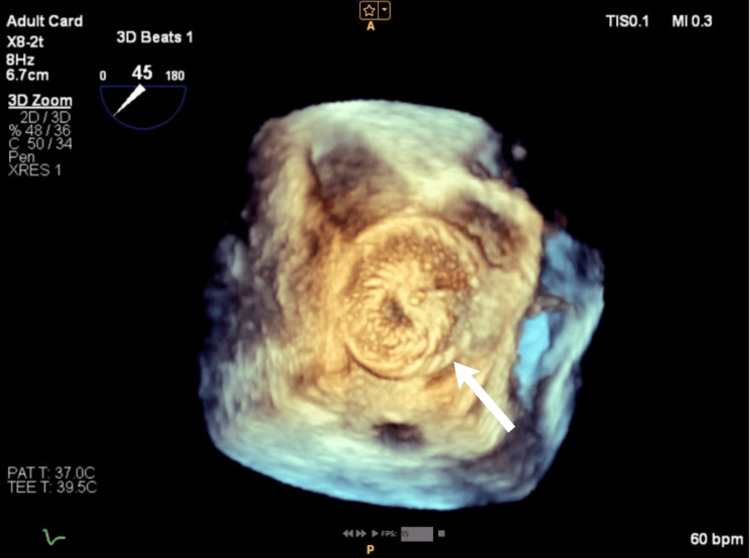
Real-time 3D TEE image of ASD II demonstrating the normal position of the device. TEE: transesophageal echocardiography; ASD II: secundum atrial septal defect

Following a multidisciplinary review and consultation with cardiac surgeons, it was decided that there was no indication for treatment of the coronary anomaly. The patient was referred for interventional closure of her ASD II, which was performed under general anesthesia with TEE guidance, via right femoral venous and right radial arterial access. Right- and left-heart catheterization with manometry demonstrated RA pressure of 7/2/4 mmHg and PA pressure of 41/11/21 mmHg. Oximetry revealed an increase in oxygen saturation from the superior vena cava (SVC) (84.4%) to the PA (90.8%), with aortic saturation of 99.7%, confirming the presence of a left-to-right shunt. Shunt quantification was performed, yielding a Qp/Qs of 1.72:1 (Qp 8.26 L/min; Qs 4.81 L/min), indicating a hemodynamically significant shunt and the indication for closure (threshold for closure >1.5:1). Coronary angiography showed a single-ostium coronary anomaly without stenosis (Figure 8). During the procedure, the size of the defect in the three standard projections was measured to be 19/18/19 mm, and transcatheter closure of ASD II with a 22 mm Amplatzer septal occluder was performed (Figures 9, 10). There was no residual left-to-right shunt immediately after device deployment.

The patient was discharged two days after the procedure in excellent clinical condition. TTE showed no residual left-to-right shunt, only mild residual right-heart dilation, good RV function (tricuspid annular plane systolic excursion (TAPSE) 30 mm), grade I-II tricuspid regurgitation with estimated RV systolic pressure (RVSP) = 30-35 mmHg, and preserved LV function (LVEF = 68%). Chest X-ray confirmed the Amplatzer occluder in optimal position and showed early regression of pulmonary hypervolemia and cardiomegaly. A cardiology visit was arranged four weeks post‐discharge, and a structured follow-up plan was put into place.

## Discussion

This case illustrates an unusual combination of two congenital heart anomalies presenting unique diagnostic and management challenges. We present the rare coexistence of a hemodynamically significant ASD II and a single-ostium coronary artery arising from the right coronary sinus with an interarterial LAD course. The principal concern in such cases is the potential for coronary compression during device closure, emphasizing the need for minimizing procedural risks with comprehensive pre-procedural evaluation to confirm that percutaneous repair can be safely performed.

In our case, TTE first demonstrated right‐heart dilation as a result of a secundum ASD with a significant L-R shunt. TEE provided further details about an aneurysmal atrial septum and a deficient anterior aortic rim. Cardiac catheterization demonstrated a left-to-right shunt with Qp/Qs 1.72:1 and mean PA pressure of 21 mmHg, supporting the indications for closure of the ASD.

Pre-procedural contrast CTA was performed to assess cardiac anatomy, and it unexpectedly revealed a single coronary ostium arising from the right sinus with LAD coursing interarterially between the RVOT and aortic root and LCx following a retroaortic route. There were no stenoses of the coronary arteries; however, the interarterial LAD carries a recognized risk of compression between the great vessels, potentially leading to myocardial ischemia or sudden cardiac death.

To better contextualize the anatomical significance of such anomalies, Angelini’s classification system offers a useful clinical framework. Angelini proposed that coronary artery anomalies should be defined and evaluated based on both their anatomic features and functional significance, rather than classifying them solely based on their origin. His model identifies six key variable anatomic features: number and location of the coronary ostia, vessel size, proximal and mid-course, arteriolar branching pattern, and termination. He further redefined the fundamental units of coronary anatomy into three “elementary arteries”: LAD, LCx, and RCA, each being characterized by their intermediate and distal segments or their dependent microvascular bed rather than by their origin or proximal course. For example, the RCA is described as a subepicardial artery situated at the right atrioventricular sulcus, providing at least one acute marginal branch to the right ventricular free wall [7]. Applying this model to our patient, the single-ostium coronary artery gave rise to three distinct elementary branches: an interarterial LAD, a retroaortic LCx, and a normally coursing RCA. Each vessel maintained its normal distal perfusion territory despite its common origin and anomalous course, providing a physiological explanation for the absence of ischemic symptoms.

Functional evaluation with a high-intensity treadmill stress test showed no chest pain, no ECG changes, and no wall-motion abnormalities. All of the above-mentioned findings supported the safety and feasibility of transcatheter ASD closure in our patient.

Some previous reports highlight the importance of defining coronary anatomy before ASD device closure. In 2012, Williams et al. described a pediatric case in which an unsuspected retroaortic course of the LCA, discovered on coronary angiography, led to abandonment of the percutaneous ASD closure due to coronary compression [10]. In contrast, Gupta et al. reported successful transcatheter closure of an ASD in an adult whose RCA followed a retroaortic path from a single ostium arising from the left coronary sinus with care to avoid impingement on the RCA [11]. Similarly, Suliman et al. documented an incidental finding of an LAD arising from the right sinus in a patient with a secundum ASD. After confirming the course and status of the anomalous vessel on CT, they proceeded with percutaneous closure using an Amplatzer device, and it was without complications [12]. Collectively, these reports demonstrate that benign anomalous coronary anatomy is not a contraindication for transcatheter device closure. They further emphasize that, even when such anomalies are identified incidentally, a comprehensive, multimodality diagnostic approach can facilitate safe and effective ASD closure.

In line with these findings, Singh et al. highlighted the role of multidetector CTA in identifying and classifying single coronary artery variants. In their case series, CTA revealed a single coronary artery arising from the left aortic sinus with RCA following a pre-pulmonic course in a patient with tetralogy of Fallot (TOF) with infective endocarditis. CTA also identified an isolated single coronary artery arising from the right aortic sinus and supported conservative management in another patient. This report highlights coronary CTA as the preferred method for assessing coronary arteries. It offers precise and non-invasive angiographic information about the origin, course, and termination of coronary anomalies due to its excellent spatial resolution and three-dimensional reconstruction capabilities [13]. In our case, CTA provided accurate 3D mapping of coronary origins and courses, confirming a single coronary ostium arising from the right sinus, with the LAD following an interarterial course, and the LCx taking a retroaortic route. These detailed anatomical insights were crucial for risk stratification and safely planning device closure of the ASD. Although CTA is not routinely performed before ASD repair, its selective use in patients with suspected or incidental coronary anomalies is a key factor in guiding treatment and ensuring safe intervention. Our case further illustrates that even with an interarterial LAD, careful anatomic mapping and negative functional testing can permit safe device closure.

In our patient, given the size of the ASD, the presence of adequate septal rims, evidence of right‐heart remodeling, and absence of inducible ischemia, percutaneous device closure was chosen over surgical repair. A multidisciplinary team discussion involving an interventional cardiologist, radiologist, and cardiothoracic surgeon weighed the risks of device‐related erosion against the higher morbidity of open-heart surgery. With no additional intracardiac anomalies identified, transcatheter closure was considered to be the best option.

The procedure was performed successfully, and post-implant imaging confirmed the device was well-positioned, with complete elimination of the shunt. The patient was discharged on aspirin for six months, together with sertraline and her known antihypertensive therapy. A structured follow-up plan was put into place to evaluate device position, residual shunt or erosion, right-heart dimensions, and pericardial findings. In addition, non-invasive coronary imaging is planned to re-evaluate the single-ostium coronary anatomy and ensure vessel patency.

## Conclusions

This case highlights the vital importance of a coordinated, multimodality imaging strategy when managing complex congenital cardiac anomalies in adults. Combining echocardiography, CTA, and functional testing allows precise risk stratification and safe procedural planning. The incidental finding of a single-ostium coronary anomaly with an interarterial LAD, although asymptomatic, substantially altered the patient’s risk profile, prompting functional testing prior to closure.

What distinguishes this case from prior reports is the coexistence of ASD II with aneurysmal atrial septum and a potentially high-risk coronary variant in an asymptomatic adult, all successfully managed through transcatheter closure. The favorable outcome reinforces that even complex congenital presentations can be treated effectively using minimally invasive techniques. Collecting similar cases in multicenter registries would help track long-term outcomes, refine surveillance strategies, and guide evidence-based therapeutic guidelines for these rare but clinically significant conditions.
